# 344. Prevalence and Impact of Post-Acute Sequelae of COVID-19 Among People Experiencing Homelessness in King County, WA Between September 2020 - May 2021

**DOI:** 10.1093/ofid/ofab466.545

**Published:** 2021-12-04

**Authors:** Emily M Scott, Sarah N Cox, Julia H Rogers, Jessica Knaster Wasse, Helen Y Chu

**Affiliations:** 1 University of Colorado, Denver, Colorado; 2 University of Washington, Seattle, Washington; 3 Public Health- Seattle & King County, seattle, Washington

## Abstract

**Background:**

Homeless shelters are high risk settings for SARS-CoV-2 transmission. People experiencing homelessness (PEH) have high rates of chronic illness, and have been disproportionately affected by COVID-19. The burden of post-acute sequelae of COVID-19 (PASC) in PEH has not been well-studied and PEH may be uniquely affected due to barriers to medical care and the potential exacerbation of existing threats to health, housing, employment, and self-care.

**Methods:**

The Seattle Flu Study conducted community-based surveillance for SARS-CoV-2 in nine homeless shelters from September 1, 2020 and May 31, 2021. Individuals with and without respiratory symptoms were tested for SARS-CoV-2 infection using a PCR assay. We completed follow-up surveys with shelter residents age ≥18 years at days 5, 10, 30 and 60+ after positive or inconclusive diagnosis with SARS-CoV-2 infection. Individuals were asked about residual symptoms, impact on activities of daily living, access to medical care, and health-related quality of life.

**Results:**

Of 51 eligible participants, 22 (43%) completed a follow-up survey, with six at day 5 or 10 survey, 11 at day 30, and 18 at day 60+. The median time from enrollment to last follow-up survey was 77 (range 49-138) days. Five (23%) participants reported at least one symptom at day 0, five (83%) at day 5 or 10, eight (73%) at day 30 and seven (39%) at day 60+ (Figure 1). Eight (36%) reported at least one symptom on a day 30 or 60+ follow up survey that interfered or prevented their daily activities. Nine (41%) received medical care at the quarantine facility. Of those with symptoms persisting beyond day 10, four (30%) received medical care outside of a medical provider at the quarantine facility.

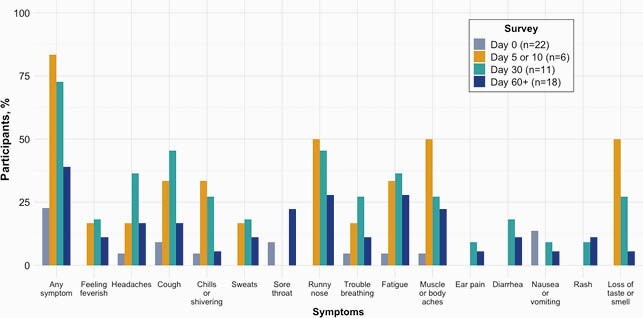

Prevalence of self-reported symptoms at Day 0 (enrollment), Day 5 or 10, Day 30, and Day 60+ in shelter residents who tested positive or inconclusive for SARS-CoV-2.

**Conclusion:**

PEH reported a high prevalence of persistent COVID-19 symptoms 30+ days after their SARS-CoV-2 detection. Few participants accessed medical care for their persistent illness. The impact of COVID-19 extends beyond acute illness and PASC may exacerbate existing challenges PEH face in health and wellbeing.

**Disclosures:**

**Helen Y. Chu, MD MPH**, **Bill and Melinda Gates Foundation** (Consultant)**Cepheid** (Research Grant or Support)**Ellume** (Consultant)**Merck** (Consultant)**Pfizer** (Consultant)**Sanofi-Pasteur** (Research Grant or Support)

